# Translocator protein (TSPO)-PET as diagnostic and monitoring tool in COVID-19 related MRI-negative brainstem encephalitis: a case report

**DOI:** 10.1007/s00415-023-11691-5

**Published:** 2023-04-03

**Authors:** Johannes Wischmann, Laura M. Bartos, Matthias Brendel, Nathalie L. Albert, Robert Forbrig, Andreas Straube, Ilias Masouris

**Affiliations:** 1 Department of Neurology, University Hospital, Ludwig-Maximilians-University (LMU), Munich, Germany; 2 Department of Nuclear Medicine, University, Ludwig-Maximilians-University (LMU), Munich, Germany; 3 Institute of Neuroradiology, University Hospital, Ludwig-Maximilians-University (LMU), Munich, Germany

**Keywords:** COVID-19, Autoimmune encephalitis, TSPO-PET, Case report

## Abstract

**Background:**

Encephalitis and myelitis have been linked to both COVID-19 vaccination and infection, causing symptoms such as reduced consciousness, mental state alterations and seizures. Remarkably, most cases do not show significant structural alterations on MRI scans, which poses a diagnostic challenge.

**Methods:**

We present the diagnostic workup and clinical course of a patient who developed a progressive brainstem syndrome two weeks after COVID-19 vaccination and subsequent infection. We used translocator protein (TSPO)-PET scans for the first time to investigate COVID-related neuroinflammation.

**Results:**

The patient developed oculomotor disorder, dysarthria, paresthesia in all distal limbs and spastic-atactic gait. CSF analysis revealed mild lymphocytic pleocytosis with normal protein levels. Brain and spinal cord MRI scans were negative, but TSPO/PET scans showed increased microglia activity in the brainstem, which correlated with the clinical course. Steroid treatment led to clinical improvement, but relapse occurred during prednisone taper after four weeks. Plasmapheresis had no significant effect; however, complete remission was achieved with cyclophosphamide and methotrexate, with normal TSPO signal ten months after onset.

**Conclusions:**

TSPO-PET can be a valuable tool in the diagnostic and therapeutic monitoring of COVID-19-related encephalitis, particularly in cases where MRI scans are negative. Aggressive immunosuppressive therapy can lead to sustained remission.

Dear Sirs,

In recent times, several cases of encephalitis related to both COVID-19 vaccination and infection have been reported [1–3]. The most common clinical features include impaired consciousness, altered mental state and seizures, which usually occur 7–14 days after vaccination or infection, while focal brainstem-related symptoms are less frequent [2, 3]. Despite this, MRI scans often show no significant changes, and cerebrospinal fluid (CSF) analysis often lacks signs of neuroinflammation on standard examination [4]. Therapeutic regimens including prednisone, plasma exchange and intravenous immunoglobulin (IVIG) have led to heterogeneous clinical outcomes ranging from complete remission to lethal courses. The neuropathogenicity mechanisms associated with COVID-19 are still under discussion [5]. Cases where SARS-CoV-2 RNA or anti-SARS-CoV-2 antibodies are detected directly in the CSF are extremely rare [2, 6]. In this study, we present a case of COVID-19-related brainstem encephalitis that was MRI-negative, and where translocator protein (TSPO)-PET was used to identify focal lesions and monitor immunosuppressive therapy.

A 34-year-old male patient was admitted to our hospital with progressive gait impairment, double vision and paresthesia of all extremities two weeks after receiving the second BioNTech/Pfizer-vaccine (BNT162b2) in December 2021. The patient had a symptomatic COVID-19 infection four days before his symptoms began, as confirmed via PCR. The patient had no notable medical or family history and had not been exposed to any potentially harmful substances. He was not taking any medication at the time of admission. Upon clinical examination, he presented with a brain stem syndrome that manifested as left-sided abducens nerve palsy, vertical gaze palsy, dysarthria, paresthesia in the distal upper and lower limbs, and spastic-atactic gait. Initial analysis of CSF showed a mild lymphocytic pleocytosis (31 cells/µl), with normal levels of protein and glucose. No specific oligoclonal bands were found in the CSF. Extensive laboratory tests of serum and CSF were conducted to rule out autoimmune and infectious diseases, and the results were normal (Table 1). Somatosensory evoked potentials of the median nerve showed normal stimulus responses at Erb´s point, N13 and N14, but a missing cortical signal (N20) on both sides (Fig. 1A). MRI of the brain and spinal cord revealed no pathological findings (Fig. 1B). The patient received methylprednisone treatment intravenously with 1 g/day for five days, followed by oral continuation and gradual dosage reduction, starting at 80 mg per day. Interestingly, TSPO-PET-scan with [^18^F]GE-180, albeit performed after prednisone-pulse therapy showed visually slightly enhanced microglia activation mainly within the medulla oblongata. However, there was no clear increase in the standardized uptake value (SUV), which was measured at 0.44 (Fig. 1E). This treatment approach led to a drastic improvement of the patient’s neurological symptoms. A follow-up after 4 weeks showed residual paresthesia in the distal upper limbs and a saccadic smooth pursuit without signs of CSF inflammation. The patient was readmitted to our hospital in May 2022 with progressive spastic gait and noticeable iatrogenic Cushing syndrome under an oral prednisone dosage of 20 mg per day. The CSF analysis was normal, and PCR of SARS-CoV-2 DNA in the CSF was negative. Brain MRI showed again no structural alterations (Fig. 1C). However, subsequent TSPO-PET showed increased microglia activation in the pons and medulla oblongata compared to the previous scan (SUV: 0.71; Fig. 1F). A whole-body FDG-PET/CT-Scan showed no signs of systemic organ involvement or malignancy. The patient received seven cycles of plasmapheresis without significant clinical improvement. Despite clinical corticoid sensitivity and due to the development of Cushing syndrome, a steroid-sparing therapy with six cycles of cyclophosphamide (500 mg/m^2^ body surface, total 6570 mg) was started in June 2022 and continued until October 2022, followed by 15 mg/week of subcutaneous methotrexate, while slowly tapering oral prednisone therapy. A follow-up evaluation in December 2022 revealed mild, non-impairing residual paresthesia of the distal upper and lower limbs. Brain MRI showed no signs of neuroinflammation or atrophy (Fig. 1D), and TSPO-PET showed no pathological tracer uptake (SUV: 0.41; Fig. 1G).

**Table 1:** Examined blood and CSF parameters

Serologic examinations	Blood count, renal and hepatic parameters, thyroid hormones, C3, C4, rheumatoid factor, ANA-, ANCA-, ENA-screen, antibodies against Ds-DNA, CCP, cardiolipin, beta-2-glykoprotein-I and serum-amyloid A
CSF parameters	Oligoclonal bands, IgM- and IgG-indices (CSF/Serum) of measles, rubella, VZV, HSV, CMV, lyme disease and syphilis
Neuroinflammatory and paraneoplastic antineural antibodies in CSF and serum	AMPA1/2, NMDA-R, DPPX, CASPR2, LGI1, GABA-a-receptor, GABA B(1/2)-R, CRMP5, AQP4, MOG, Neurexin-3-alpha, ERC1, Spez612, AP3B2, Contactin1, Neurofascin 155, Neurofascin 186, AT1A3, KCNA2, dopamine-receptor 2, Hu, Ri, ANNA-3, Yo, Myelin, Ma/Ta, GAD65, ampiphysin, CARPVIII, glycine-receptor, mGluR1, mGluR5, Rho, GTPase activation protein 26, ITPR1, Homer 3, Recoverin, Neurochondrin, GluRD2, Flotillin-1/2, IgLON5, anti-purkinje-cell-IgG and Zic4
Viral and bacterial CNS pathogens in serum (S) and CSF	DNA-PCR of H. influenza (CSF), L. monocytogenes (CSF), N. meningitides (CSF), S. agalactiae (CSF), S. pneumonia (CSF), C. neoformans (CSF), E. coli (CSF), mycoplasma pneumonia (CSF), CMV (CSF), enteroviruses (CSF), HSV-1/2 (CSF), HHV-6 (CSF), HPV (CSF), VZV (CSF), HBV (S), HCV (S) and HIV (S)

**Fig. 1:**
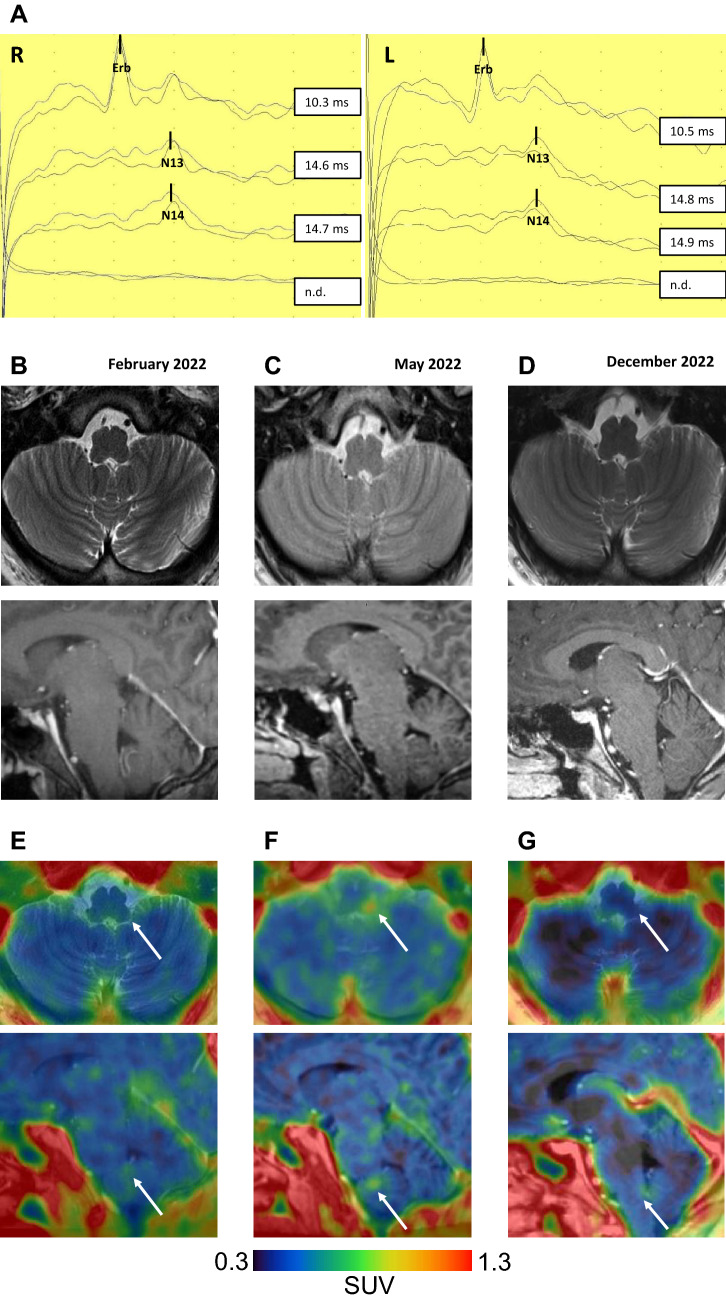
Neurophysiology and -imaging of the patient. **A** Somatosensory evoked potentials of the right (R) and left (L) median nerve and corresponding measured latencies. Stimulus responses were acquired at Erb’s point (Erb), C7 (N13), C2 (N14) and cortical (N20). Horizontal interval between dots: 50 ms. Vertical interval between dots: 2 µV. n.d. = not detectable. **B**–**D** Axial T2 and sagittal contrast enhanced T1 MRI-sequences of the brain with TSPO-PET scan overlays. **E**–**G** Images were acquired at baseline in February after prednisone-pulse therapy (left column), clinical deterioration in May (middle column) and after cyclophosphamide therapy in December 2022 (right column). Tracer enhancement (white arrows) was measured by standardized uptake value (SUV) in a tracer-dosage- and bodyweight-dependent manner. The SUV are absolute values without normalization to a reference region. All TSPO-images were displayed with a uniform SUV-range of 0.3–1.3 to allow visual comparability. Faint focal tracer uptake was detected visually mainly in sagittal view within the dorsal medulla oblongata at baseline (SUV: 0.44; **E**). Clinical deterioration correlated with progressive enhanced microglia activation within the dorsal pons and medulla oblongata (SUV: 0.71; **F**). After cyclophosphamide therapy, no pathological tracer uptake was measured anymore (SUV: 0.41; **G**)

We herein report a case of brainstem encephalitis temporally associated with a BioNTech/Pfizer COVID-19 vaccination and subsequent COVID-19 infection. Unlike previously reported cases of COVID-related encephalitis, which typically featured rather diffuse symptoms like encephalopathy or delirium, our patient exhibited a distinct focal brainstem syndrome. Although initial CSF analysis showed signs of inflammation, subsequent laboratory testing ruled out other infectious and non-infectious causes. Multiple MRI scans did not reveal any structural lesions corresponding to the patient’s symptoms, which is consistent with previous reports of MRI-negative COVID-19-related encephalitis and myelitis [3, 4, 7]. Repeated TSPO/PET scans, however, identified a lesion in the dorsal pons and medulla oblongata. Moreover, the TSPO/PET signal closely matched the clinical syndrome anatomically and correlated well with the severity and course of the patient's neurological symptoms under immunosuppressive therapy. Notably, in the initial TSPO-scan, there was a focal enhancement in the dorsal medulla, visually distinct in sagittal view, with only a faint increase in SUV-value. This is possibly attributed to the fact that the patient received prednisone-pulse therapy prior to the initial TSPO-imaging. Remission of symptoms coincided with clearance of tracer uptake. Thus, we believe that TSPO/PET could aid in the diagnosis and therapy monitoring of COVID-19-related encephalitis, particularly in cases where MRI results are negative.

There is an ongoing debate regarding a possible direct neuropathogenicity of COVID-19 and a susceptibility of the medulla oblongata to the virus [8]. Notably, patients who died from COVID-19 have been found to exhibit significantly higher microglial activation in the brainstem compared to controls [5]. SARS-CoV-2 is believed to cause its neuropathogenic effects primarily indirectly by activating complex immunogenic pathways [9]. Specifically, neuroinflammation may be mediated by the innate immune system after microglia activation triggered by SARS-CoV-2 infection or vaccination, which involves proinflammatory cytokines such as IL-1ß, IL-6, and TNF-alpha [2, 10, 11]. TSPO-PET can detect TSPO expression in activated microglia, which supports the hypothesized pathophysiology of COVID-19-related encephalitis [9]. We cannot distinguish between vaccination and infection as the main trigger of our patient’s neurological manifestations. However, it can be speculated whether the subsequent COVID-19 infection provided an additional immunogenic stimulus to priory susceptible microglia, as the concept of primed microglia is well established [10]. Further studies are warranted to profoundly examine COVID-19-related neuropathogenicity.

Severe cases and clinical deterioration under immunosuppressive therapy are not uncommon in COVID-related encephalitis [4]. Due to the lack of standardized therapeutic guidelines and the diversity of applied treatments in published cases, systematic comparison of therapeutic regimens can be challenging. In our case, the patient responded significantly to steroids and cyclophosphamide, and maintenance therapy with methotrexate resulted in ongoing clinical stabilization. In cases of COVID-related encephalitis, aggressive immunosuppressive therapy may be necessary even after achieving remission with prednisone to prevent a relapse.

In conclusion, TSPO-PET is a promising diagnostic tool for detection of focal lesions and suitable to monitor the therapy response in COVID-related MRI-negative focal encephalitis. Cyclophosphamide and methotrexate can induce stable remission.

## References

[1] Zuhorn F (2021). Postvaccinal encephalitis after ChAdOx1 nCov-19. Ann Neurol.

[2] Siow I (2021). Encephalitis as a neurological complication of COVID-19: a systematic review and meta-analysis of incidence, outcomes, and predictors. Eur J Neurol.

[3] Ellul MA (2020). Neurological associations of COVID-19. Lancet Neurol.

[4] Ariño H (2022). Neuroimmune disorders in COVID-19. J Neurol.

[5] Poloni TE (2021). COVID-19-related neuropathology and microglial activation in elderly with and without dementia. Brain Pathol.

[6] Lou JJ (2021). Neuropathology of COVID-19 (neuro-COVID): clinicopathological update. Free Neuropathol.

[7] Abrams RMC (2021). MRI negative myelopathy post mild SARS-CoV-2 infection: vasculopathy or inflammatory myelitis?. J Neurovirol.

[8] Serrano GE et al (2021) Mapping of SARS-CoV-2 brain invasion and histopathology in COVID-19 Disease. medRxiv 2021.02.15.21251511

[9] Vivash L, O'Brien TJ (2016). Imaging microglial activation with TSPO PET: lighting up neurologic diseases?. J Nucl Med.

[10] Giannotta G, Giannotta N (2018). Vaccines and Neuroinflammation. Int J Pub Health Safe.

[11] Mishra R, Banerjea AC (2021). SARS-CoV-2 spike targets USP33-IRF9 axis via exosomal miR-148a to activate human microglia. Front Immunol.

